# Additive effects of 10-year exposures to PM_2.5_ and NO_2_ and primary cancer incidence in American older adults

**DOI:** 10.1097/EE9.0000000000000265

**Published:** 2023-08-01

**Authors:** Yaguang Wei, Mahdieh Danesh Yazdi, Tszshan Ma, Edgar Castro, Cristina Su Liu, Xinye Qiu, James Healy, Bryan N. Vu, Cuicui Wang, Liuhua Shi, Joel Schwartz

**Affiliations:** a Department of Environmental Health, Harvard T.H. Chan School of Public Health, Boston, Massachusetts; b Program in Public Health, Department of Family, Population, and Preventive Medicine, Renaissance School of Medicine at Stony Brook University, Stony Brook, New York; c Gangarosa Department of Environmental Health, Rollins School of Public Health, Emory University, Atlanta, Georgia; d Department of Epidemiology, Harvard T.H. Chan School of Public Health, Boston, Massachusetts

**Keywords:** Air pollution, Primary cancers, Causal modeling, Additive effects

## Abstract

Epidemiologic evidence on the relationships between air pollution and the risks of primary cancers other than lung cancer remained largely lacking. We aimed to examine associations of 10-year exposures to fine particulate matter (PM_2.5_) and nitrogen dioxide (NO_2_) with risks of breast, prostate, colorectal, and endometrial cancers.

**Methods:**

For each cancer, we constructed a separate cohort among the national Medicare beneficiaries during 2000 to 2016. We simultaneously examined the additive associations of six exposures, namely, moving average exposures to PM_2.5_ and NO_2_ over the year of diagnosis and previous 2 years, previous 3 to 5 years, and previous 6 to 10 years, with the risk of first cancer diagnosis after 10 years of follow-up, during which there was no cancer diagnosis.

**Results:**

The cohorts included 2.2 to 6.5 million subjects for different cancers. Exposures to PM_2.5_ and NO_2_ were associated with increased risks of colorectal and prostate cancers but were not associated with endometrial cancer risk. NO_2_ was associated with a decreased risk of breast cancer, while the association for PM_2.5_ remained inconclusive. At exposure levels below the newly updated World Health Organization Air Quality Guideline, we observed substantially larger associations between most exposures and the risks of all cancers, which were translated to hundreds to thousands new cancer cases per year within the cohort per unit increase in each exposure.

**Conclusions:**

These findings suggested substantial cancer burden was associated with exposures to PM_2.5_ and NO_2_, emphasizing the urgent need for strategies to mitigate air pollution levels.

**What this study adds**

Although air pollution has been identified as a carcinogen for lung cancer, the relation between air pollution and other primary cancers remained largely unquantified. We estimated the simultaneous associations of 10-year exposures to fine particulate matter (PM_2.5_) and nitrogen dioxide (NO_2_) with the risks of breast, prostate, colorectal, and endometrial cancers among the national Medicare cohort between 2000 and 2016. Our results showed that PM_2.5_ and NO_2_ exposures increased the risks of colorectal and prostate cancers. At low exposure levels, we observed substantially larger associations between most exposures and the risks of all cancers.

## Introduction

Outdoor air pollution is a leading contributor to the global burden of disease.^1^ Outdoor air pollution is a complex mixture of particles and irritant gases such as organic aerosols, heavy metals, and nitrogen dioxide (NO_2_).^2^ Once inhaled, those pollutants can penetrate deeper into the lungs and are carried by the bloodstream throughout the body. A recent global review showed that chronic exposure to air pollution can affect almost every organ in the body, causing a variety of diseases and conditions.^3^

In 2013, the International Agency for Research on Cancer identified air pollution as a group 1 carcinogen for lung cancer.^4^ For other cancers, genotoxicity and molecular biomarker data from experimental studies demonstrated that air pollution is associated with altered gene expressions, inflammation, and oxidative stress in tissues other than the lung, indicating potential links between air pollution and the risk of developing various types of cancers.^5–8^ Epidemiologic evidence on air pollution and other cancers remained largely lacking but was growing substantially in recent years.^9–12^ Nevertheless, most existing studies had relatively short follow-up periods and evaluated exposures from one up to a few years before cancer diagnosis.^13–16^ Due to cancer’s long latency period, those studies might not be able to adequately identify critical windows of susceptibility or capture the full effect. Besides, previous studies were limited to small unrepresentative samples, which was further complicated by the heterogeneity in cancer.^17–19^ Additionally, previous studies had reported risks on a multiplicative scale, which obscured the actual size of the risk and are more difficult to interpret compared with the additive effect estimates.^20^ Further, the competing risk of death was pervasive but commonly overlooked, leading to potential overestimation of the effect size.^21^

We aimed to examine the simultaneous associations of 10-year exposures to fine particulate matter (PM_2.5_) and NO_2_, the two major primary pollutants emitted directly from fossil fuel and biomass combustion sources, on the risks of breast, prostate, colorectal, and endometrial cancers, for national Medicare beneficiaries ≥65 years of age with continuous enrollment in the Fee-for-Service (FFS) program and in the Medicare Parts A and B. To provide a better sense of the risk posed by the pollutants, we estimated additive associations and translated them to the numbers of new cancer cases per unit increase in each exposure, accounting for the competing risk of death.

## Methods

### Study population and design

We obtained the Medicare denominator file and the Medicare Chronic Conditions Warehouse (CCW) from the Centers for Medicare and Medicaid Services.^22^ The Medicare denominator file contained enrollment records and demographic information, including sex, race/ethnicity, age, Medicaid enrollment status (a marker of low socioeconomic status), ZIP code of residence, and date of death (if any), for each Medicare beneficiary. Age, Medicaid enrollment status, and ZIP code of residence were updated annually. The CCW contained the date of the first occurrence of cancer diagnosis for each cancer type across all available claims in the FFS and the Medicare Parts A (inpatient) and B (outpatient, physician service, home health, etc.). To reduce the chance of missing cancer diagnoses, we restricted the cohort to beneficiaries with continuous enrollment in FFS and in both Part A and Part B throughout the follow-up period. This was because if a beneficiary spent any time in the Medicare Advantage Plans (Part C), or only one of Parts A or B during the study period, there was the possibility that the cancer claims were not available for CCW.^23^

For each cancer, we constructed a separate cohort in which each beneficiary was followed since 2000 or entering the study. To allow for the assessment of the exposure effects over the 10 years before cancer diagnosis, we implemented an inclusion criterion that required a minimum follow-up period of 10 years after enrollment, during which no cancer diagnosis occurred. That is, we only included beneficiaries who were cancer-free for the initial 10 years of follow-up. By applying the 10-year cancer-free inclusion criterion, we also ensured that the first identified cancer diagnosis was an incidence case. If a beneficiary had been diagnosed with cancer before enrolling in Medicare, we believed they would have had a claim record for physician visit, treatment, hospitalization, etc. with cancer diagnosis within the first 10 years after study entry. After that, beneficiaries were censored at the first occurrence of cancer diagnosis, death, or the end of the study in 2016, whichever occurred earliest. We restricted the cohort to females for breast and endometrial cancers and males for prostate cancer.

This study was approved by the Institutional Review Boards at the Harvard T.H. Chan School of Public Health and Emory University.

### Exposures

We predicted daily average outdoor PM_2.5_ and NO_2_ levels at 1-km^2^ grid cells across the contiguous US using geographically weighted regressions that ensembled predictions from random forests, gradient boosting, and neural network.^24–27^ The predictions integrated multiple data sources including satellite data, land-use variables, monitoring data, chemical transport model simulations, etc. The ensembled models demonstrated better predictive performance than that of the individual machine learners with 10-fold cross-validated R-squared values of 0.89 for annual PM_2.5_ predictions and 0.84 for annual NO_2_ predictions. This performance was in held-out monitors not used for model fitting. Based on these predictions at 1-km^2^ grid cells and ZIP code shapefiles provided by the Environmental Systems Research Institute (ESRI), we estimated concentrations at ZIP codes by averaging the predictions at grids whose centroids were inside the polygonal area for general ZIP codes, or assigning the prediction at the nearest grid for other ZIP Codes that did not have polygon representations, such as apartment buildings, military bases, or post offices.^28^

The ZIP code-level concentrations were considered as proxy exposure measurements and were linked to each beneficiary in each year based on their residential ZIP codes. For each beneficiary in each year, we examined three exposure windows: the moving average exposure over that year and the previous two years (lag 0–2 years), the moving average over the previous 3 to 5 years (lag 3–5 years), and the moving average over the previous 6 to 10 years (lag 6–10 years).

### Community-level covariates

We included a variety of annual, ZIP code-level covariates in the analysis as potential confounders and effect modifiers. The percent of ever smokers, average body mass index (BMI), and number of active medical doctors per 1000 people were derived from the Behavioral Risk Factor Surveillance System and the Area Health Resources Files.^29,30^ The percent White, percent Black, percent below poverty, percent rental housing, percent education below high school, and median household income were derived from the Decennial Census and the American Community Survey.^31–33^ The distance from the centroid of each ZIP code to the nearest hospital was calculated based on the ESRI’s hospital distribution files, which was considered as a proxy for average distance to the nearest hospital.^34^ The percent of Medicare beneficiaries with diabetes who had a lipid-panel test in a year and the percent beneficiaries with at least one ambulatory doctor’s visit in a year were derived from the Dartmouth Health Atlas.^35^ Rurality, as measured by population density, was derived from the NASA Socioeconomic Data and Applications Center.^36^ The Normalized Difference Vegetation Index (NDVI), a measure of greenness that was associated with cancer incidence,^37^ was obtained from the NASA’s Moderate Resolution Imaging Spectroradiometer Land data products.^38^ The national Area Deprivation Index (ADI), a composite measure of neighborhood disadvantage level, was obtained from the Neighborhood Atlas website.^39^

### Statistical analysis

We had six exposures of interest, specifically lag 0 to 2, lag 3 to 5, and lag 6 to 10 exposures to PM_2.5_ and NO_2_. For each cancer and each exposure, to obtain the independent effect estimate, we employed an inverse probability weighting (IPW)-based additive model in which the other five exposures were adjusted as confounders.^20,40^ The analysis consisted of two stages: a design stage where the inverse probability weight (IPW) of each person-year’s exposure was estimated to provide a scalar summary that can be used to eliminate confounding and competing risk through weighting, and an analysis stage where the additive association of the exposure with the cancer risk was estimated.

In the first stage, the IPW (*sw*_*i*_) for person-year *i* was estimated by


$$sw_{i}=\frac{K\left(x_{i}-\bar{x}\right)}{K\left(x_{i}-\hat{g}\left(x_{i}|c_{i}\right)\right)}\times \frac{\hat{g}\left(D_{i}=0|x_{i}\right)}{\hat{g}\left(D_{i}=0|x_{i},\;\;\;c_{i}\right)},$$

where *K* (·) represented a kernel density estimator; *x*_*i*_ the exposure level; $\bar{x}$ the average exposure across all person-years; *c*_*i*_ a set of confounders including the other five exposures, individual risk factors (sex [male or female], race/ethnicity [White, Black, or other], age group after initial 10-year cancer-free follow-up [75–84 or ≥85 years], and Medicaid enrollment status [yes or no]), and community-level covariates as delineated above; *D*_*i*_ the indicator of death (0 or 1); and $\hat{g}(\cdot )$ a gradient boosting machine (GBM) prediction model with a gaussian loss function.^41^ Overall, the above function incorporated the stabilized IPWs for both confounding and competing risk of death. The use of nonparametric algorithms of kernel density and GBM had superior performance over parametric models in terms of bias and flexibility: the kernel density function relaxed the strong assumption of distributional form of the exposure residuals, and the GBM captured all possible nonlinearity and interactions of covariates and was unaffected by the potential autocorrelation.^42,43^ If the assumptions underlying the propensity score were met, this would provide a causal model.^40^

In the analysis stage, we fitted the following linear probability model weighted by the IPWs obtained in the previous stage to estimate the additive association of the exposure with the absolute cancer risk,


$$E\left(Y_{i}=1|x_{i}\right)=\beta_{0}+\beta_{1}x_{i},$$

where *Y*_*i*_ was the outcome of cancer diagnosis (0 or 1). Given the estimated association *β*_1_, we further estimated the annual number of new cancer cases attributed to a unit increase in the exposure by


$$\text{Annual}\;\text{number}\;\text{of}\;\text{new}\;\text{cancer}\;\text{cases}={\hat{\beta }}_{1}\times \frac{N}{\text{Duration}\;\;\text{of}\;\text{study}},$$

where *N* was the total number of person-years of follow-up (including those of beneficiaries with cancer diagnoses in the first 10 years of follow-up) and the duration of study was 17 years.

To examine whether the associations remained at low exposure levels, for PM_2.5_, we repeated the whole analysis for person-years with exposure levels at lag 0 to 2, lag 3 to 5, and lag 6 to 10 years all below 10 µg/m^3^; for NO_2_, we repeated the whole analysis for person-years with exposure levels at lag 0 to 2, lag 3 to 5, and lag 6 to 10 years all below 20 parts per billion (ppb). We further examined the associations at stricter exposure levels, specifically the newly updated World Health Organization air quality guidelines ([AQG]; 5 µg/m^3^ for PM_2.5_ and 10 ppb for NO_2_).^44^

To examine whether certain subpopulations and communities experienced higher cancer risk in the impact of air pollution, we conducted separate analysis for each subgroup by age, sex (for colorectal cancer only), race/ethnicity, ever enrollment in Medicaid, and upper or lower quartile of community-level percent of ever smokers, average BMI, population density, and ADI.

## Results

The cohorts had 3.8 million participants for breast cancer, 6.5 million for colorectal cancer, 4.0 million for endometrial cancer, and 2.2 million for prostate cancer. The cohorts predominantly consisted of Whites (>90%) and beneficiaries aged between 75 and 84 years (>80%) after initial 10-year cancer-free follow-up (Table 1). Beneficiaries excluded from the cohort due to a cancer diagnosis, death, or end of the study within the first 10 years after the Medicare enrollment had larger population sizes, higher numbers of cancer diagnoses, and lower proportions of White (Table S1; http://links.lww.com/EE/A234). Over the study period between 2000 and 2016, the annual concentrations of PM_2.5_ averaged 9.8 µg/m^3^ and annual NO_2_ averaged 17.3 ppb (Table 2). For general ZIP Codes that had polygon representations, the average distance from a ZIP Code centroid to its boundary was ≈7.3 km; the fifth percentile corresponded to a distance of 1.2 km, while the 95th percentile represented a distance of 17.5 km.

**Table 1.** Demographic characteristics of the constructed Medicare cohorts.

	Breast cancer	Colorectal cancer	Endometrial cancer	Prostate cancer
Number of individuals (%)	3,756,831 (100)	6,457,863 (100)	4,044,008 (100)	2,161,156 (100)
Number of person-years	55,133,844	94,243,120	59,429,929	31,113,440
Number of cancer diagnoses (%)	90,421 (2.4)	68,456 (1.0)	21,660 (0.5)	80,615 (3.7)
Sex				
Male (%)	0	2,459,690 (38.1)	0	2,161,156 (100)
Female (%)	3,756,831 (100)	3,998,173 (61.9)	4,044,008 (100)	0
Race				
White (%)	3,416,371 (91.0)	5,902,528 (91.4)	3,682,920 (91.1)	1,989,780 (92.1)
Black (%)	206,382 (5.5)	328,257 (5.1)	220,178 (5.4)	90,702 (4.2)
Other^a^ (%)	134,078 (3.5)	227,078 (3.5)	140,910 (3.5)	80,674 (3.7)
Age group after initial 10-year cancer-free follow-up (years)				
75–84 (%)	3,105,175 (82.7)	5,511,808 (85.4)	3,353,113 (82.9)	1,918,776 (88.8)
≥85 (%)	651,656 (17.3)	946,055 (14.6)	690,895 (17.1)	242,380 (11.2)
Ever enrolled in Medicaid (%)	486,759 (13.0)	665,057 (10.3)	513,226 (12.7)	146,367 (6.8)

^a^Other indicates Asian, Hispanic, Native American, Pacific Islander, and multiracial individuals.

**Table 2.** Distributions of annual concentrations of PM_2.5_ and NO_2_ at ZIP codes among all years in the contiguous US.

	PM_2.5_ (µg/m^3^)	NO_2_ (ppb)
Minimum	0.0	0.0
10th percentile	5.8	7.1
25th percentile	7.6	9.7
Median	9.8	15.0
Mean	9.8	17.3
75th percentile	11.8	21.9
90th percentile	14.0	31.1
Maximum	30.9	127.6

Results of the main analysis and low-level analysis are shown in Table 3. For breast cancer, exposures to PM_2.5_ at lag 0 to 2 and lag 3 to 5 years were statistically and significantly associated with decreased cancer risk, while at lag 6 to 10 year, the association was insignificant. Exposures to NO_2_ at lag 0 to 2, lag 3 to 5, and lag 6 to 10 years were all associated with increased breast cancer risk. In the low-level analysis in which the analysis was restricted to exposure levels below 10 µg/m^3^ for PM_2.5_ and below 20 ppb for NO_2_, both pollutants across the lag periods were associated with increased cancer risk. Notably, in the below-AQG analysis in which the analysis was restricted to exposure levels below 5 µg/m^3^ for PM_2.5_ and below 10 ppb for NO_2_, substantially larger positive associations were observed for both pollutants across the lag periods. These additive associations were translated to hundreds to thousands of newly diagnosed breast cancer cases in each year within the cohort. As an example, each 1-µg/m^3^ increase in lag 0 to 2 PM_2.5_ was associated with 3,291 (95% confidence interval [CI] = 2,280, 4,302) new breast cancer diagnoses per year, and each 1-ppb increase in lag 6 to 10 NO_2_ was associated with 809 (95% CI = 548, 1,071) new breast cancer diagnoses per year among the cohort.

**Table 3.** Absolute increases (and 95% CIs) in the risk of cancer diagnosis and the annual number of new cancer cases per unit increase in PM_2.5_ and NO_2_.

	Breast cancer		Colorectal cancer		Endometrial cancer		Prostate cancer	
	Absolute increase in the risk of cancer diagnosis	Annual number of new cancer cases	Absolute increase in the risk of cancer diagnosis	Annual number of new cancer cases	Absolute increase in the risk of cancer diagnosis	Annual number of new cancer cases	Absolute increase in the risk of cancer diagnosis	Annual number of new cancer cases
Main analysis^a^								
PM_2.5_, lag 0–2 years	−0.0024% (−0.0038%, −0.0010%)	−78 (−125, −32)	0.0047% (0.0040%, 0.0054%)	261 (220, 301)	0.0003% (−0.0004%, 0.0009%)	10 (−13, 33)	0.0072% (0.0048%, 0.0097%)	132 (87, 178)
PM_2.5_, lag 3–5 years	−0.0026% (−0.0038%, −0.0013%)	−83 (−125, −42)	0.0040% (0.0033%, 0.0046%)	219 (183, 255)	−0.0005% (−0.0011%, 0.0001%)	−18 (−38, 3)	0.0067% (0.0045%, 0.0089%)	123 (82, 163)
PM_2.5_, lag 6–10 years	0.0006% (−0.0004%, 0.0017%)	21 (−14, 56)	0.0041% (0.0035%, 0.0046%)	225 (195, 255)	−0.0005% (−0.0010%, −0.0001%)	−19 (−36, −2)	0.0112% (0.0094%, 0.0131%)	205 (171, 240)
NO_2_, lag 0–2 years	0.0018% (0.0014%, 0.0021%)	57 (45, 69)	0.0005% (0.0003%, 0.0007%)	29 (18, 39)	0.0000% (−0.0001%, 0.0002%)	1 (−5, 7)	0.0035% (0.0029%, 0.0041%)	64 (52, 75)
NO_2_, lag 3–5 years	0.0014% (0.0011%, 0.0017%)	46 (35, 56)	0.0009% (0.0007%, 0.0010%)	48 (39, 58)	0.0000% (−0.0002%, 0.0001%)	−2 (−7, 4)	0.0041% (0.0035%, 0.0046%)	74 (64, 85)
NO_2_, lag 6–10 years	0.0012% (0.0009%, 0.0015%)	39 (29, 49)	0.0003% (0.0002%, 0.0005%)	18 (9, 26)	−0.0001% (−0.0003%, 0.0000%)	−5 (−10, 0)	0.0047% (0.0041%, 0.0052%)	85 (75, 95)
Low-level analysis^b^								
PM_2.5_, lag 0–2 years	0.0177% (0.0136%, 0.0218%)	575 (441, 708)	0.0069% (0.0050%, 0.0089%)	383 (276, 491)	0.0031% (0.0012%, 0.0050%)	109 (44, 174)	0.0380% (0.0314%, 0.0446%)	695 (575, 816)
PM_2.5_, lag 3–5 years	0.0075% (0.0036%, 0.0113%)	242 (116, 368)	0.0078% (0.0060%, 0.0097%)	434 (332, 536)	0.0006% (−0.0011%, 0.0024%)	22 (−40, 84)	0.0310% (0.0248%, 0.0371%)	566 (453, 680)
PM_2.5_, lag 6–10 years	0.0158% (0.0121%, 0.0195%)	512 (392, 632)	0.0065% (0.0048%, 0.0083%)	362 (265, 458)	0.0000% (−0.0017%, 0.0017%)	1 (−58, 61)	0.0228% (0.0168%, 0.0287%)	417 (308, 525)
NO_2_, lag 0–2 years	0.0079% (0.0065%, 0.0092%)	255 (211, 299)	0.0033% (0.0026%, 0.0040%)	182 (144, 221)	0.0030% (0.0023%, 0.0036%)	104 (82, 125)	0.0057% (0.0034%, 0.0080%)	105 (63, 147)
NO_2_, lag 3–5 years	0.0043% (0.0030%, 0.0057%)	141 (97, 185)	0.0008% (0.0001%, 0.0014%)	42 (4, 80)	0.0009% (0.0002%, 0.0015%)	30 (8, 52)	0.0053% (0.0031%, 0.0076%)	97 (56, 139)
NO_2_, lag 6–10 years	0.0052% (0.0040%, 0.0064%)	169 (130, 208)	0.0001% (−0.0005%, 0.0007%)	8 (−26, 41)	−0.0005% (−0.0010%, 0.0001%)	−17 (−36, 2)	0.0115% (0.0094%, 0.0135%)	210 (173, 247)
Below-AQG analysis^c^								
PM_2.5_, lag 0–2 years	0.1015% (0.0703%, 0.1326%)	3,291 (2,280, 4,302)	0.0631% (0.0490%, 0.0772%)	3,499 (2,717, 4,281)	0.0265% (0.0123%, 0.0407%)	926 (430, 1,423)	0.1986% (0.1525%, 0.2448%)	3,635 (2,791, 4,480)
PM_2.5_, lag 3–5 years	0.0537% (0.0207%, 0.0868%)	1,743 (672, 2,814)	0.0140% (−0.0004%, 0.0285%)	777 (−24, 1,579)	0.0280% (0.0136%, 0.0425%)	980 (475, 1,484)	0.1583% (0.1100%, 0.2066%)	2,897 (2,014, 3,780)
PM_2.5_, lag 6–10 years	0.0673% (0.0330%, 0.1017%)	2,184 (1,070, 3,298)	0.0521% (0.0367%, 0.0676%)	2,890 (2,034, 3,746)	−0.0130% (−0.0282%, 0.0021%)	−455 (−985, 75)	0.1040% (0.0540%, 0.1541%)	1,904 (989, 2,819)
NO_2_, lag 0–2 years	0.0345% (0.0269%, 0.0420%)	1,118 (872, 1,364)	0.0180% (0.0142%, 0.0218%)	997 (785, 1,209)	0.0121% (0.0086%, 0.0156%)	423 (300, 546)	0.0568% (0.0442%, 0.0693%)	1,039 (809, 1,268)
NO_2_, lag 3–5 years	0.0188% (0.0109%, 0.0267%)	609 (353, 866)	0.0000% (−0.0039%, 0.0039%)	0 (−214, 215)	0.0043% (0.0007%, 0.0080%)	152 (24, 279)	0.0192% (0.0065%, 0.0318%)	351 (119, 583)
NO_2_, lag 6–10 years	0.0249% (0.0169%, 0.0330%)	809 (548, 1,071)	0.0067% (0.0026%, 0.0107%)	371 (146, 595)	0.0040% (0.0003%, 0.0078%)	141 (10, 272)	0.0409% (0.0276%, 0.0542%)	748 (505, 992)

^a^Main analysis was conducted among the entire cohort.

^b^For PM_2.5_, the low-level analysis was conducted among person-years with exposure levels at lag 0–2 years, lag 3–5 years, and lag 6–10 years all below 10 µg/m^3^; for NO_2_, the low-concentration analysis was conducted among person-years with exposure levels at lag 0–2 years, lag 3–5 years, and lag 6–10 years all below 20 ppb.

^c^For PM_2.5_, the below-guideline analysis was conducted among person-years with exposure levels at lag 0–2 years, lag 3–5 years, and lag 6–10 years all below 5 µg/m^3^; for NO_2_, the below-guideline analysis was conducted among person-years with exposure levels at lag 0–2 years, lag 3–5 years, and lag 6–10 years all below 10 ppb.

For colorectal cancer, both pollutants across the lag periods were associated with increased cancer risk. The effect sizes for PM_2.5_ were larger than those for NO_2_. In the low-level analysis, associations for PM_2.5_ were slightly larger than those for the main analysis, and for NO_2_ the associations attenuated with increasing lag periods. In the below-AQG analysis, substantially larger associations were observed for both pollutants, which were translated to hundreds to thousands of newly diagnosed colorectal cancer cases per year among the cohort.

For endometrial cancer, most associations were not statistically significant across the entire exposure ranges. However, the lag 6 to 10 PM_2.5_ exposure was marginally and significantly associated with decreased cancer risk. In the low-level and below-AQG analyses, the associations for both pollutants attenuated with increasing lag periods.

For prostate cancer, all the exposures were associated with increased cancer risk across the entire exposure ranges. In the low-level analysis, all the effect sizes were larger compared with those in the full exposure ranges. In the below-AQG analysis, the associations became considerably larger, which were translated to hundreds to thousands of newly diagnosed prostate cancer cases per year among the cohort.

**Figure 1.**
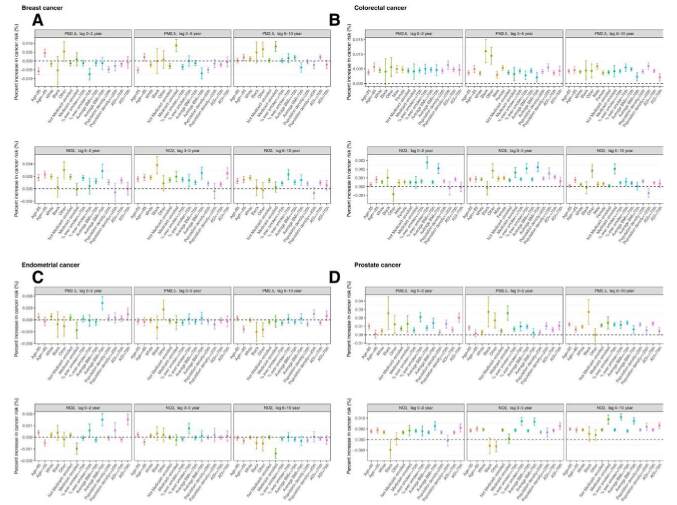
Absolute increases (and 95% CIs) in the risk of cancer diagnosis and the annual number of new cancer cases per unit increase in PM_2.5_ and NO_2_ for each subgroup of individual and community-level characteristics. Subgroups of community-level characteristics were divided by upper or lower quartiles.

Susceptibility among subpopulations was mostly inconsistent across exposures and cancer types (Figure 1). For breast cancer, those who ever enrolled in Medicaid experienced higher risk associated with PM_2.5_ exposures over lag 3 to 5 and lag 6 to 10 years, and those who lived in ZIP codes with higher BMI (>75th percentile) had higher risk for NO_2_ exposures at lag 0 to 2 and lag 3 to 5 years. For colorectal cancer, those who lived in ZIP codes with higher rate of ever smokers (>75th percentile) or average BMI (>75th percentile) had higher risk associated with NO_2_ exposures at lag 0 to 2 and lag 3 to 5 years. For endometrial cancer, those who lived in ZIP codes with higher average BMI (>75th percentile) were at a higher cancer risk associated with lag 0 to 2 PM_2.5_ and lag 0 to 2 NO_2_ exposures. For prostate cancer, Blacks were at a higher cancer risk associated with PM_2.5_ exposures across lag 0 to 2, lag 3 to 5, and lag 6 to 10 years, and those who lived in ZIP codes with higher average BMI (>75th percentile) had higher risk associated with NO_2_ exposures across lag 0 to 2, lag 3 to 5, and lag 6 to 10 years.

## Discussion

In this cohort study of millions of Medicare beneficiaries across the contiguous US, we found clear evidence that chronic exposures to PM_2.5_ and NO_2_ increased the risks of colorectal and prostate cancers in a window of 10 years before diagnosis, consistent with the most recent epidemiological studies.^11,13,14,16,17,45–47^ Compared with previous studies, our study controlled for the competing risk of death, used propensity score and an additive model that provided marginal estimates of effect sizes, and found stronger associations at lower concentrations, including below the newly updated WHO guidelines. In the main analysis, when considering the mutually adjusted and independent additive effect estimates, the combined effects over three exposure windows were substantial: for colorectal cancer, an unit reduction in long-term PM_2.5_ and NO_2_ would prevent at least 705 and 95 annual incident cases within the cohort, respectively; for prostate cancer, an unit reduction in long-term PM_2.5_ and NO_2_ would prevent at least 460 and 223 annual cases within the cohort, respectively. Given that the effect sizes were considerably larger at exposure levels below the newly updated WHO guidelines, further reducing the pollution levels would achieve greater health and economic benefits by preventing more cancers. In contrast, there was little evidence observed in our main analysis supporting the relevance of PM_2.5_ and NO_2_ exposures to endometrial cancer risk, consistent with the Japan’s National Cancer Registry study.^12^ However, in the low-level and below-AQG analyses, the larger associations suggested that there may be stronger relations between lower levels of PM_2.5_ and NO_2_ exposures and the risk of endometrial cancer, and the concentration-response relations may follow a nonlinear pattern in an additive scale.

For breast cancer, the results can be interpreted as associations for postmenopausal breast cancer risk, considering that the Medicare cohort consisted of individuals aged 75 years or older at the time of diagnosis. In accord with Crouse et al.^48^ and Poulsen et al.^13^ who investigated the postmenopausal women, we found that NO_2_ was negatively associated with breast cancer incidence. Evidence for PM_2.5_ was less clear. Consistent with several large cohort studies,^15,16,18,49–51^ we detected both positive and negative association for PM_2.5_ across the entire exposure range. The mixed associations in our study and the previous studies may be due to the heterogeneous changes in chemical compositions and sources of PM_2.5_.^19^ The difference in PM_2.5_ compositions may also explain the more varying results for PM_2.5_ than those for NO_2_. However, when restricting the analysis to lower exposure levels, where the compositions of local PM_2.5_ remained fairly stable,^52^ we found strongly and consistently adverse impacts of PM_2.5_. Clearly, future studies with data on PM_2.5_ compositions are needed to explore the relations of specific chemical components with breast cancer risk. Similar to endometrial cancer, the substantially larger associations in the low-level and below-AQG analyses indicated that the effect of air pollution on breast cancer risk may be more pronounced at lower exposure levels.

Few studies had explored the potential impact of individual- and community-level factors on the susceptibility of cancer to air pollution.^53^ In this study, we found inconsistent subgroup differences across exposures and cancer types, possibly due to the complex etiology of different cancers.^9^ However, we found suggestive evidence indicating a disproportionately higher risk of all the cancers associated with NO_2_ exposures among individuals residing in communities with higher average BMI, suggesting that a lack of access to healthy food and limited opportunities for physical activities may contribute to an increased cancer susceptibility to the adverse effect of NO_2_.^54^ The finding of greater susceptibility of the Medicaid enrollees and Blacks to the PM_2.5_-induced cancer risks was also important in terms of improving health equity. Blacks had been exposed to higher levels of air pollution,^55^ so the larger effect sizes resulted in an even greater injustice.

Our study had several strengths. First, the inclusion of millions of participants with long-term follow-up allowed for investigating the exposures later in life, from up to 10 years before cancer diagnosis, with ample statistical power. Second, the independent and additive associations made it possible to combine them to obtain the overall estimates for a window of 10 years before cancer diagnosis. As a result, adding up the individual effect estimates for each pollutant was a straightforward and valid approach that provided a better sense of its long-term impact. Third, the use of GBM and kernel density estimator achieved a high level of flexibility and accuracy for estimating IPW, making the results robust against any observed confounding bias. Fourth, accounting for competing risk avoided the associations being overestimated.

This study also had limitations. First, restricted by available data sources, we could not capture individual-level risk factors such as smoking status and BMI, but used community-level smoking rate and average BMI as surrogates, which may lead to confounding bias. For breast cancer, it is important to note that reproductive factors and the use of hormone therapy were not available for the analysis.^56^ For colorectal cancer, the analysis did not account for alcohol consumption, a recognized risk factor.^57^ While these factors may not be directly associated with air pollution exposures and therefore may not confound the association between air pollution and cancer incidence, incorporating them in future research can still contribute to obtaining more robust effect estimates. Second, the use of ZIP code-level exposures was subject to measurement error, especially for NO_2_, which has greater spatial variability.^58^ However, the larger ZIP codes were generally in less populated and rural areas, where the spatial variability in NO_2_ exposure may be smaller than in urban areas, where the ZIP codes are smaller. This may limit, but not eliminate, the exposure error. A recent simulation study suggested that correcting for exposure measurement error would lead to larger effect estimates.^59^ Third, participants in our study were restricted to Medicare beneficiaries with continuous enrollment in both FFS program and Part A and B, which may limit generalizability to the total elderly population. Fourth, restricting the analysis to only beneficiaries who were cancer-free for the initial 10 years of follow-up may introduce selection bias, considering the demographic differences between the analysis group and excluded group. There was a possibility that the beneficiaries who developed cancer during the initial 10 years might be more susceptible to the effects of air pollution. In this case, overlooking those beneficiaries could lead to the associations being underestimated.

## Conclusions

Our study of the national Medicare cohort investigated additive associations of 10-year exposures to PM_2.5_ and NO_2_ with the risks of breast, prostate, colorectal, and endometrial cancers. We found that exposures to PM_2.5_ and NO_2_ were associated with increased risks of colorectal and prostate cancers, but were not associated with endometrial cancer risk. NO_2_ was associated with a decreased risk of breast cancer, while the association for PM_2.5_ remained inconclusive. At lower exposure levels, in particular below the newly updated WHO AQG, we observed substantially larger associations between most exposures and the risks of all cancers. The observed additive associations resulted in a substantial number of newly diagnosed cancer cases per year within the cohorts, ranging from hundreds to thousands for each unit increase in exposure. These findings suggested substantial cancer burden associated with exposures to PM_2.5_ and NO_2_, emphasizing the urgent need for strategies to mitigate air pollution levels.

## Conflicts of interest statement

The authors declare that they have no they have no conflicts of interest with regard to the content of this report.

This work was supported by the National Institutes of Health grants R01ES032418 and P30ES000002.

The Medicare data are available upon request to the Centers for Medicare and Medicaid Services. The grid-level PM_2.5_ data are available at https://doi.org/10.7927/0rvr-4538. The grid-level NO_2_ data are available at https://doi.org/10.7927/f8eh-5864. The ZIP code-level PM_2.5_ and NO_2_ are available at https://doi.org/10.7927/9yp5-hz11. Other covariate data are publicly available with sources described in the article.

## ACKNOWLEDGMENTS

Y.W. and J.S. did conceptualization. Y.W., M.D.Y., E.C., C.S.L., and L.S. did data curation. Y.W. did formal analysis. Y.W., T.M., X.Q., J.H., B.N.V., C.W., L.S. did methodology. J.S. did supervision. Y.W. wrote the original draft. Y.W., M.D.Y., T.M., E.C., C.S.L., X.Q., J.H., B.N.V., C.W., L.S., and J.S. did review and editing.

## Supplementary Material
