# Supplementary material for: Additive effects of 10-year exposures to PM2.5 and NO2 and primary cancer incidence in American older adults
Source: Environ Epidemiol. 2023 Aug 1;7(4):e265. doi: 10.1097/EE9.0000000000000265 (PMC10402937; doi:10.1097/EE9.0000000000000265)
Supplement: Supplementary file 1 [file ee9-7-e265-s001.docx]

**Supplementary Material**

Table S1. Demographic characteristics of Medicare beneficiaries who had continuous enrollment in the Fee-for-Service program and in Part A & B throughout the follow-up period, but were excluded from the analysis due to a cancer diagnosis within the first 10 years after the Medicare enrollment, death, or end of the study in 2016.

|  | Breast cancer | Colorectal cancer | Endometrial cancer | Prostate cancer |
| --- | --- | --- | --- | --- |
| Number of individuals (%) | 5529694 (100) | 10116335 (100) | 5666035 (100) | 4401955 (100) |
| Number of person-years | 27420107 | 49892601 | 28129090 | 21440560 |
| Number of cancer diagnoses (%) | 312249 (5.6) | 194194 (1.9) | 68790 (1.2) | 375013 (8.5) |
| Sex |  |  |  |  |
| Male (%) | 0 | 4437201 (43.9) | 0 | 4401955 (100) |
| Female (%) | 5529694 (100) | 5679134 (56.1) | 5666035 (100) | 0 |
| Race |  |  |  |  |
| White (%) | 4818564 (87.1) | 8756531 (86.6) | 4935299 (87.1) | 3793312 (86.2) |
| Black (%) | 357808 (6.5) | 637682 (6.3) | 366656 (6.5) | 260563 (5.9) |
| Other* (%) | 353322 (6.4) | 722122 (7.1) | 364080 (6.4) | 348080 (7.9) |
| Age group at study entry (years) |  |  |  |  |
| 65–74 (%) | 5002755 (90.5) | 9286602 (92.8) | 5146304 (90.8) | 4089109 (92.9) |
| 75–84 (%) | 311362 (5.6) | 524282 (5.2) | 298636 (5.3) | 228042 (5.2) |
| ≥85 (%) | 215577 (3.9) | 305451 (3.0) | 221095 (3.9) | 84804 (1.9) |
| Ever enrolled in Medicaid (%) | 656337 (11.9) | 1034956 (10.2) | 670230 (11.8) | 360552 (8.2) |

* Other indicates Asian, Hispanic, Native American, Pacific Islander, and multiracial individuals.
